# Myoepithelial tumor with *EWSR1-PBX3* fusion and rearrangement in rib and soft tissue: a rare case report

**DOI:** 10.3389/fonc.2026.1776713

**Published:** 2026-02-27

**Authors:** Junting Yang, Jie Liu, Tingting Zhao, Shuwei Yu, Boxiukang Zhang, Yangcan Li, Hongling Wang

**Affiliations:** 1 Department of Clinical Laboratory, Qingdao University Medical College Affiliated Yantai Yuhuangding Hospital, Yantai, China; 2 School of Medicine, Xizang Minzu University, Xianyang, China; 3 Department of Radiology, Qingdao University Medical College Affiliated Yantai Yuhuangding Hospital, Yantai, China; 4 Department of Pathology, The First Hospital of Changsha, Changsha, China; 5 Department of Material Supply, Qingdao University Medical College Affiliated Yantai Yuhuangding Hospital, Yantai, China

**Keywords:** *EWSR1-PBX3*, molecular diagnosis, myoepithelial tumor, rib tumor, soft tissue tumor

## Abstract

Myoepithelial tumor (MET) is rare and heterogeneous. We present a case of a MET occurring in the rib and the soft tissue surrounding it. Further, the tumor has been reported to contain both *EWSR1-PBX3* fusion and rearrangement. A 52-year-old male presented with worsening low back pain. Imaging showed osteolytic destruction of the left twelfth rib accompanied by a soft-tissue mass. Histopathology revealed spindle and oval tumor cells with mild atypia. The entity was positive for S-100, EMA, SMA and calponin by immunohistochemistry but negative for cytokeratin. Testing through next-generation sequencing (NGS) verified the presence of the *EWSR1-PBX3* fusion and rearrangement. After surgical resection and post-operative radiotherapy, the patient remained disease-free for 2 years. The presence of *EWSR1-PBX3* fusion and rearrangement in METs of bone and soft tissue is diagnostically significant. It is critical that molecular testing should be performed by clinicians, notably when distinguishing it from synovial sarcoma and other closely related entities.

## 1 Introduction

Myoepithelial tumors (METs) found in bone and soft tissue are uncommon mesenchymal tumors that the World Health Organization (WHO) categorizes as having uncertain differentiation. The main subtypes include mixed tumor, myoepithelioma, and myoepithelial carcinoma (1). Mixed tumors have tubular differentiation akin to their salivary gland equivalents. Myoepitheliomas mainly consist of spindle-shaped or oval myoepithelial cells with minimal atypia, while moderate to marked atypia is a characteristic of myoepithelial carcinoma.

While most myoepithelial tumors express epithelial markers like cytokeratin and/or epithelial membrane antigen (EMA) along with S-100 protein, there is a significant amount of variability among individual cases (2, 3). Because of their marked morphological and immunophenotypic heterogeneity, molecular genetic profiling has emerged as a valuable method for accurate characterization. For example, Salivary gland and skin lesions often show alterations in *PLAG1* and *HMGA2* genes, while approximately 50% of myoepithelial tumors in bone, soft tissue, and visceral organs possess recurrent fusions related to *EWSR1* or *FUS*. Currently, the confirmed fusion partners include *POU5F1, PBX1, ZNF444, ATF1*, and *PBX3* (4–6).

The *EWSR1–PBX3* fusion was first detected in skin lesions but is now more commonly found in soft tissue and intraosseous myoepithelial tumors. Typically, tumors containing this fusion exhibit minimal or no cytokeratin expression and are generally related to a good prognosis (6–8). There are few studies on intraosseous METs that involve *EWSR1-PBX3* fusion and reported cases most frequently involve the maxilla, ilium, tibia, and fibula (5, 6).

This study presents a unique case of myoepithelial tumor involving both the rib and adjacent paraspinal soft tissue, characterized by the coexistence of *EWSR1-PBX3* fusion and rearrangement. As far as we know, this is among the initial documented instances of a paraspinal soft-tissue myoepithelioma invading a nearby rib with this distinct genetic change.

### 2.1 Clinical findings

**Figure 1:**
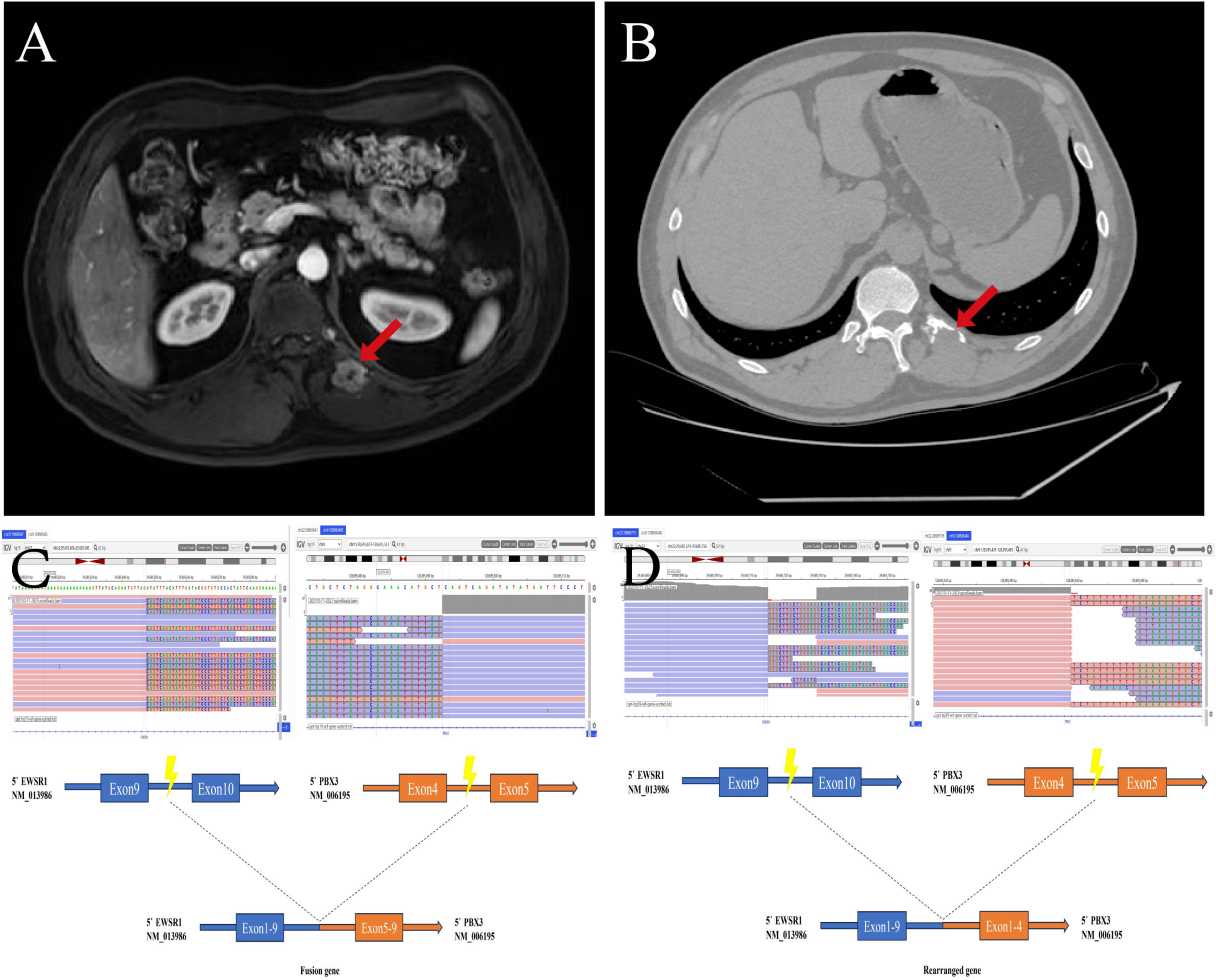
Imaging examination and molecular features. **(A)** MRI in axial plane, **(B)** Computed tomography in axial plane, **(C)** The fusion site was at exon 9 of the EWSR1 and exon 5 of the PBX3 gene. **(D)** The rearrangement site was at exon 9 of the EWSR1 and exon 4 of the PBX3 gene.

A 52-year-old male presented with lower back pain for three years, without any obvious cause, which worsened in 2023. Imaging revealed a 1.9 × 2.0 cm mass at the left costovertebral junction, involving the posterior aspect of the twelfth rib and the adjacent T12 vertebral body, accompanied by osteolytic destruction (**Figures 1A, B**). PET-CT demonstrated osteolysis of the left posterior twelfth rib with increased metabolic activity showing increased FDG uptake (maximum SUV 7.4) and an indistinct boundary with adjacent musculature. Laboratory tests showed a notably elevated serum neuron-specific enolase level of 31.9 ng/ml (reference:0–17 ng/ml), while other tumor markers were within normal limits.

### 2.2 Histopathology and immunohistochemistry

**Figure 2:**
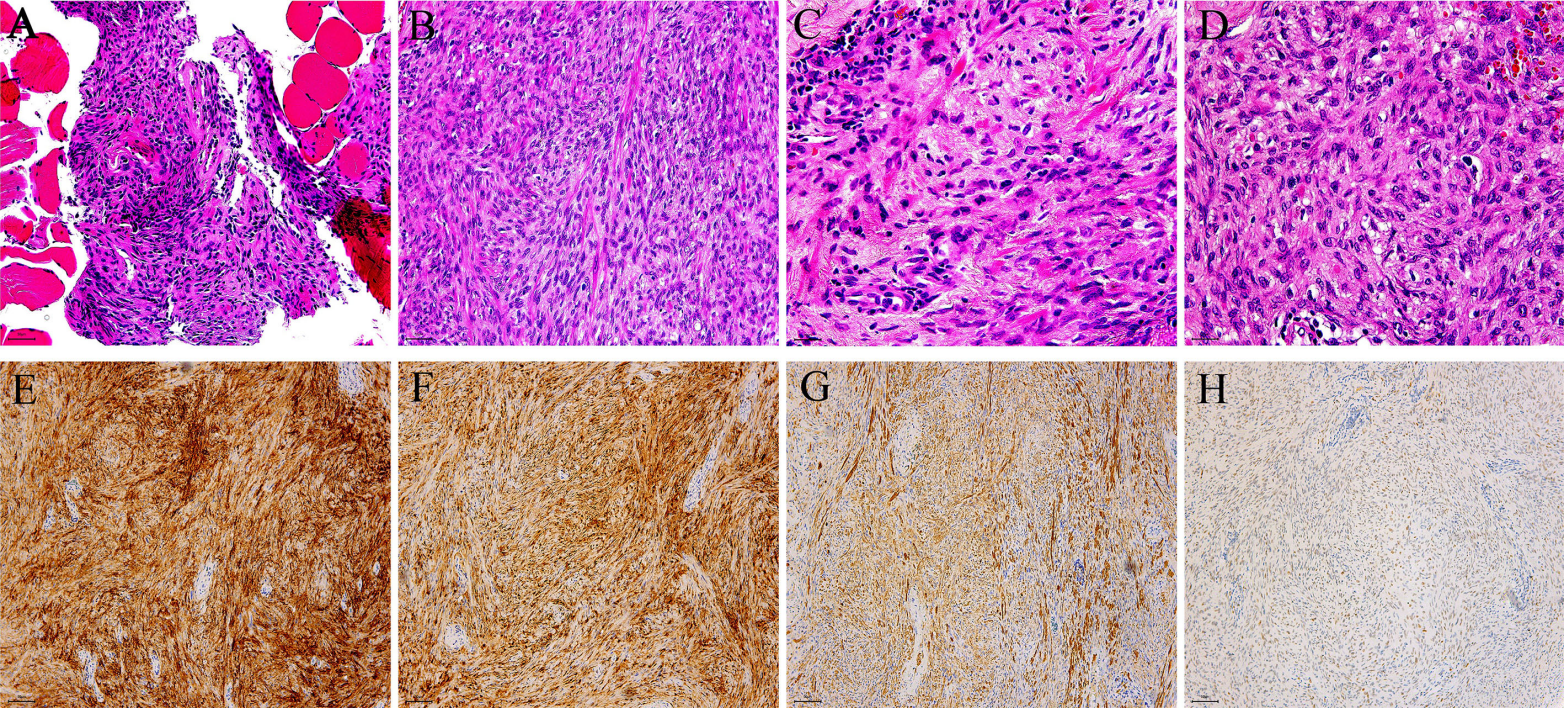
Photomicrographs of hematoxylin and eosin (H&E)-stained slides are shown as follows: **(A)** rib at 200×, **(B)** soft tissue at 200×, **(C)** rib at 400×, and **(D)** soft tissue at 400×. Images **(E)** to **(H)** represent positive immunohistochemical staining for EMA, S100, SMA, and TLE1, respectively.

As shown in **Figure 2**, histological examination under low magnification revealed a nodular tumor with poorly demarcated borders and a partially encapsulated profile. The lesion featured zones of hemorrhage and coagulative necrosis, intermingled with a scattered lymphocytic infiltrate and focal vasculitic changes. It was moderately hypercellular, with tumor cells forming distinctive storiform and fascicular architectures (**Figures 2A, B**). Under high power lens examination, the cellular population was composed of spindel, oval, and epithelioid cells that displayed mild cytological atypia without significant mitotic figures (**Figures 2C, D**).

Immunohistochemical analysis of paraffin sections revealed that in bone tissue, vimentin, calponin, S-100, EMA, and SMA were moderately to strongly positive (**Figures 2E–G**), whereas CK, CD34, SOX10, Bcl-2, CR, desmin, and STAT6 were negative, with a Ki-67 index of approximately 2%. In adjacent soft tissue, EMA, SMA, S-100, calponin, CD99, CD56, ALK, and CD117 were moderately to strongly positive, while CD34, CR, CK, and BCOR were negative, with a Ki-67 index of around 3%. Additional immunohistochemical staining demonstrated positive immunoreactivity for TLE1 (**Figure 2H**).

### 2.3 Next-generation sequencing analysis

To refine the diagnosis and explore potential therapeutic targets, DNA-based targeted next-generation sequencing (NGS) was performed using the Illumina NextSeq 550Dx platform with a panel (Genecast Biotechnology Co., Ltd.) covering 769 cancer-related genes. The sequencing data quality was rigorously controlled: the average deduplicated depth across all targeted regions was 1,058×, and the Q30 score was 96.86%. Both positive and negative control samples included in the run passed all quality metrics. Breakpoints were identified at *EWSR1* chr22:g.29685647 (NM:013986) and *PBX3* chr9:g.128695493 (NM:006195), resulting in an in-frame fusion of *EWSR1* exon 9 to *PBX3* exon 5, which forms the primary *EWSR1-PBX3* (E9;P5) fusion gene with an allelic frequency of 8.95% (**Figure 1C**). A second rearrangement was detected between *EWSR1* chr22:g.29685715 and *PBX3* chr9:g.128695449, generating *EWSR1-PBX3* (E9;P4) with an allelic frequency of 5.95% (**Figure 1D**). In addition, *ARID1B* p.H172delH mutation was observed. Microsatellite instability testing indicated a microsatellite-stable (MSS) profile, and the tumor mutational burden (TMB) was detected as <1.00 mutations/Mb (low), with a quantile value of 10.23%.

### 2.4 Treatment and follow-up

**Figure 3:**
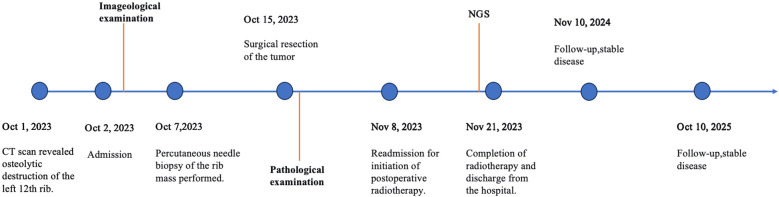
Timeline of the main events.

The left twelfth rib, left transverse process of the twelfth thoracic vertebra (T12), head of the left 12th rib, the associated left T12 costotransverse joint and adjacent soft tissue were resected. Adjuvant radiotherapy was administered one month post-operation. The timeline of the main events is outlined in **Figure 3**. At the two-year follow-up, the patient remained free of local recurrence and distant metastasis.

## 3 Discussion and conclusion

While myoepithelial tumors are frequently documented in scientific literature, instances involving the *EWSR1-PBX3* fusion are extremely uncommon. This report presents a distinctive case that demonstrates both the *EWSR1-PBX3* fusion and rearrangement. In the absence of RNA evidence, we propose that the *EWSR1::PBX3 (E9;P5)* rearrangement represents the candidate driver event, constituting a fusion gene, whereas the *EWSR1::PBX3 (E9;P4)* rearrangement, due to its 5’-to-5’ configuration, likely represents a non-productive structural rearrangement without expression of a functional fusion transcript. In previously reported cases, around fifty percent of soft-tissue and bone myoepithelial tumors contain *EWSR1* gene fusions. The *EWSR1-PBX3* fusion partner was first identified by Agaram et al. in 2014 in two intraosseous and one soft-tissue tumor (5). Suurmeijer et al. subsequently reported 10 additional cases harboring *EWSR1-PBX3* fusion in METs (6). Later, Yun and Haglund described intraosseous lesions in the metatarsal and ulna, respectively (9, 10). Gandhi et al. reported six further cases, demonstrating a spectrum of osseous myoepithelial tumors ranging from benign to malignant (11). To date, approximately twenty cases of bone myoepithelial tumors featuring the *EWSR1-PBX3* fusion have been described in various skeletal and soft tissue sites, including the tibia, fibula, phalanges, navicular, radius, and ulna. The present case represents the first reported example arising in the rib.

Microscopically, the tumor displayed pinkish nodular tissue with hemorrhage and coagulative necrosis. It consisted of bland oval-to-spindle cells with eosinophilic cytoplasm and scattered perivascular lymphocytes. Immunohistochemically, both bone and soft tissue components were positive for S100, EMA, and Calponin, but negative for cytokeratin. The *EWSR1-PBX3* fusion is most commonly found in cutaneous syncytial myoepithelioma (CSM), which is a benign tumor noted for its syncytial growth pattern and the co-expression of EMA and S-100, along with the repetitive occurrence of the *EWSR1-PBX3* fusion. Compared with the broader group of myoepithelial tumors, CSM displays a more uniform immunophenotypic and genetic profile (12–14). A small subset of myoepithelial tumors, however, arises in soft tissue or bone. The distinction between benign and malignant tumors harboring this fusion is often based on mitotic count in hematoxylin and eosin (H&E)-stained sections. Malignant lesions typically show marked nuclear atypia, prominent nucleoli, increased mitotic activity and areas of necrosis. Although most osseous METs with *EWSR1-PBX3* fusion are benign, a minor subset exhibits malignant features.

Morphologic and immunohistochemical evaluation remains the cornerstone for identifying METs; however, due to their marked heterogeneity and complexity, detection of EWSR1-PBX3 fusion provides substantial value for accurate clinical classification. Misdiagnosis can occur in challenging cases—for example, as Haglund noted, diffuse and strong SATB2 staining may lead to an erroneous diagnosis of osteosarcoma (10). Similarly, TLE1 positivity in our case could mimic synovial sarcoma; although TLE1 is considered a sensitive and specific marker for synovial sarcoma, its biological role remains uncertain (15, 16). In our case, the initial misdiagnosis was partly due to the absence of confirmatory SS18-SSX fusion testing, which is a critical diagnostic marker for synovial sarcoma (17). Subsequent NGS analysis not only failed to detect any SS18-related alterations but conclusively identified an EWSR1 rearrangement, thereby definitively establishing the diagnosis of myoepithelial tumor. Therefore, molecular characterization of EWSR1-PBX3 fusion is essential for accurate diagnosis of myoepithelial tumors of bone and soft tissue. In addition, we also tested positive for an ARID1B p.H172delH mutation. ARID1B is known to promote cancer progression primarily by disrupting chromatin remodeling, impairing DNA repair, and dysregulating the cell cycle (18). ARID1B is frequently mutated in cancers such as breast, ovarian, and lung carcinomas (19–21); however, its role in myoepithelial tumors remains unstudied. We hypothesize that it may lead to dysregulation of chromatin remodeling, compromise normal gene expression control, and potentially contribute to carcinogenesis in this context.

To summarize, we present a compelling case involving *EWSR1-PBX3* fusion and rearrangement located in the twelfth rib and surrounding soft tissue. This finding contributes additional insights into the molecular mechanisms underlying myoepithelial tumors in both bone and soft tissue.

## Data Availability

The original contributions presented in the study are included in the article/supplementary material. Further inquiries can be directed to the corresponding author.
